# Biological Control: A Major Component of the Pest Management Program for the Invasive Coconut Scale Insect, *Aspidiotus rigidus* Reyne, in the Philippines

**DOI:** 10.3390/insects11110745

**Published:** 2020-10-30

**Authors:** Billy Joel M. Almarinez, Alberto T. Barrion, Mario V. Navasero, Marcela M. Navasero, Bonifacio F. Cayabyab, Jose Santos R. Carandang, Jesusa C. Legaspi, Kozo Watanabe, Divina M. Amalin

**Affiliations:** 1 Biology Department, College of Science, De La Salle University, Taft Avenue, Manila 1004, Philippines; billy.almarinez@dlsu.edu.ph (B.J.M.A.); albertotbarrion1114@gmail.com (A.T.B.); jose.santos.carandang@dlsu.edu.ph (J.S.R.C.VI); 2 Biological Control Research Unit, Center for Natural Science and Environmental Research, De La Salle University, Taft Avenue, Manila 1004, Philippines; 3 National Crop Protection Center, University of the Philippines Los Baños, Los Baños, Laguna 4031, Philippines; mvnavasero@gmail.com (M.V.N.); cely_navasero@yahoo.com.ph (M.M.N.); bfcayabyab@yahoo.com (B.F.C.); 4 Center for Medical, Agricultural and Veterinary Entomology, United States Department of Agriculture—Agricultural Research Service, Tallahassee, FL 32308, USA; jesusa.legaspi@ars.usda.gov; 5 Center for Marine Environmental Studies, Ehime University, Matsuyama, Ehime 790-8577, Japan; watanabe.kozo.mj@ehime-u.ac.jp

**Keywords:** *Comperiella calauanica*, Encyrtidae, *Aspidiotus rigidus*, Diaspididae, coconut scale insect, host density-dependent parasitism, parasitization levels, natural biological control

## Abstract

**Simple Summary:**

Major outbreaks of the coconut scale insect (CSI), *Aspidiotus rigidus*, occurred in the Philippines, between 2010 and 2016 in the Southern Tagalog region of Luzon Island, and from 2017 to 2020 in the Zamboanga Peninsula, Mindanao Island. Contribution of parasitization by *Comperiella calauanica* to the management of the outbreaks needed quantification. Our assessments found very high percent parasitization by *C. calauanica*, which occurred naturally in Southern Tagalog. Findings were similar in the Zamboanga Peninsula, where mass-reared *C. calauanica* from Southern Tagalog were released for biological control. Significant decreases in the population of CSI were recorded in 1- to 2-year periods, 2014 to 2016 in Southern Tagalog, and 2018 to 2020 in the Zamboanga Peninsula, with parallel findings of recovery by infested coconut palms. Compared to the integrated pest management (IPM) program in Southern Tagalog, that in the Zamboanga Peninsula relied less on chemical control, and no major climatic disturbance was recorded in the region. Our findings strongly suggest that biological control has been a very important factor in the management of *A. rigidus*. Rearing facilities for *C. calauanica* as biological control agent should be established and maintained, especially in the different regions, to enable quick response to new areas of CSI invasion in the country.

**Abstract:**

The coconut scale insect, *Aspidiotus rigidus* Reyne, caused a major pest outbreak in coconut plantations and stands in the Southern Tagalog region of Luzon Island in the Philippines between 2010 and 2015. To determine if parasitism by *Comperiella calauanica* Barrion, Almarinez and Amalin, a native encyrtid, could have been a factor in the eventual management of the outbreak by 2015, we estimated and assessed its parasitization levels on *A. rigidus* colonies on field-collected samples from selected points in three provinces in the Southern Tagalog Region across three sampling periods. We observed that *C. calauanica* consistently occurred only in areas where *A. rigidus* populations occurred, with high parasitization levels in the Southern Tagalog sites from 2014 to 2015. Results of correlation and regression of total scale count against parasitized scale count suggest putative host density-dependent parasitism by *C. calauanica* in the field. A marked decrease in the abundance of *A. rigidus* was recorded concurrently with visually observable recovery of coconut trees from the third quarter of 2014 up to the second quarter of 2016. Similar results of significant reduction in *A. rigidus* populations concurrent with high percent parasitization by mass-reared and released *C. calauanica* were found in the Zamboanga Peninsula from 2018 to 2020. Our findings and observations altogether suggest that host-specific parasitization by *C. calauanica* effected biological control, which may have contributed to the eventual management of the *A. rigidus* outbreak in the Southern Tagalog Region, and also in the Zamboanga Peninsula where similar recovery of coconut trees were observed within a year after inoculative releases of *C. calauanica*.

## 1. Introduction

The coconut palm, *Cocos nucifera* L., is one of the top agricultural commodities in the Philippines. Coconut, together with banana, pineapple, and coffee, leads other locally produced crops in terms of export value [1]. Coconut plantations cover more than 3 million hectares of land area in the country, which has been recognized as among the top producers of coconut in the world [2].

Infestation of an armored scale insect (Hemiptera: Diaspididae) on coconut trees was first observed in 2009 in the province of Batangas on Luzon Island, and had reached devastating outbreak levels in the following years from 2010 to 2014. This diaspidid, known locally as “*cocolisap*”, was previously recognized by local authorities as *Aspidiotus destructor* Signoret until a more detailed study revealed its correct species identity as *Aspidiotus rigidus* Reyne [3]. *A. rigidus* was first reported as a subspecies of *A. destructor* by Reyne [4] in Sangi Island in Northern Sulawesi, Indonesia. This insect feeds on the lower surfaces of the leaflets of coconut fronds, consequently impairing photosynthesis and transpiration, and possibly leading to death of the tree in 6 months or less [3]. The outbreak that spread from Batangas to other parts of Southern Luzon led to the well-documented devastation in coconut plantations, recognized only sometime in mid-2014 as a national emergency by the Philippine government. Trunk injection of a systemic pesticide, the neonicotinoid dinotefuran, was carried out as a principal part of emergency control measures implemented to address the outbreak. By the end of 2014, the *A. rigidus* infestation in Southern Luzon was declared to have declined to a non-outbreak level by the Philippine Coconut Authority (PCA) after observation of recovery of coconut trees subjected to the control measures. The recovery was attributed to the effects of Typhoon Rammasun and chemical control by trunk injection of dinotefuran [5]. Effective insecticides may reduce *A. rigidus* populations in the field, but it is important to select appropriate chemical agents, and observe proper timing and application methods to protect beneficial insects. Prior to the *A. rigidus* outbreak in the Philippines, concerns over the use of dinotefuran and other neonicotinoids have already been raised internationally due to their high toxicity to bees and pollinators [6,7].

Biological control refers to the use of natural enemies to manage or suppress populations of a pest, through conservation of natural enemies, or augmentation by release of mass-reared natural enemies [8]. A high degree of host specificity, which implies minimal risk of non-target effects, is preferred for natural enemies being considered as candidate agents for biological control of a pest [9,10]. For an invasive pest species, classical biological control is considered to be among the most important control measures [11]. Parasitoids, when compared to predators, tend to have a stronger effect on pest mortality when used as biological control agents [10]. Hymenopteran parasitoids have been considered the most important group of insects as far as biological control is concerned [12]. Parasitic wasps most frequently employed in biological control usually belong to the families Braconidae, Ichneumonidae, Eulophidae, Pteromalidae, Encyrtidae, and Aphelinidae, while dipteran parasitoids belong to Tachinidae, Phoridae, or Cryptochetidae [10]. The use of biological control agents, particularly parasitic Hymenoptera, has been reported to be important in the control of scale insect pests. For instance, the coconut scale insect, *Aspidiotus destructor*, in the Philippines is controlled by the hymenopteran parasitoid, *Aphytis lingnanensis* Compere (Aphelinidae) [3]. In Pakistan, *A. destructor*, is also reported to be attacked by two parasitoids, *Aphytis melinus* DeBach and *Anagyrus* sp. (Encyrtidae) [13]. For the closely related diaspidid species, *A. rigidus*, *Comperiella unifasciata* Ishii (Encyrtidae) was among the reported natural enemies in Indonesia [4,14].

The genus *Comperiella*, with type species *C. bifasciata*, consists of species of dark colored, tiny wasps that are parasitic on a variety of diaspidids [15]. Females of *Comperiella* spp. insert their eggs in the covered bodies of the host at varying host life stages depending on species. The larvae, upon emerging from the eggs, feed inside the host while developing in a holometabolous manner, effectively killing the host prior to emergence of the adult parasitoid. In 1927, *C. unifasciata* was released in Sangi Island, Indonesia to control the outbreak of *A. rigidus* [4]. However, it did not control the outbreak due to low mean parasitization rate that was recorded only at 12% [4,14]. In the Philippines, *C. bifasciata* was reported to have been introduced in 1935 from Japan for the biological control of the citrus pest, *Aonidiella aurantii* Maskell [16]. No native species of *Comperiella* had been identified in the country until 2014 [17,18]. This *Comperiella* sp. has been described as a new species, and named *C. calauanica* by Barrion et al. [19]. *C. calauanica* has also been confirmed in field and laboratory observations as being highly parasitic to *A. rigidus*, with initial records showing up to 80% parasitization, suggesting the potential of this encyrtid as a biological control agent against *A. rigidus* [18].

The direct association of *C. calauanica* on *A. rigidus* was confirmed from the initial field survey of natural enemies of *A. rigidus* [18]. In the current study, we describe the parasitism efficiency of *C. calauanica* on *A. rigidus* based on field assessment of parasitization and host abundance, and visual observation of recovery of scale-infested coconut trees in the field. Additionally, the host density-dependence of *C. calauanica* is assessed. Findings from our data and observations suggest the putative role of the encyrtid in the control of the diaspidid pest in important coconut-planting areas in the Philippines, particularly in the islands of Luzon and Mindanao.

## 2. Materials and Methods

### 2.1. Field Survey and Sample Collection

Field surveys were done on separate dates from 2014 to 2016 for Southern Tagalog, Luzon Island, and from 2018 to 2020 for Zamboanga Peninsula, Mindanao Island. The survey dates were based on the reported occurrence of the coconut scale insects in the areas. The sample collection method for Southern Tagalog was developed by the research team. However, for Zamboanga Peninsula the Rapid Ground Assessment (RGA) method developed by the Philippine Coconut Authority (PCA) (2018, unpublished), an attached agency of the Philippine Department of Agriculture, was followed to be in line with the government protocol. Details of both methods are described in the following section for the estimation of the parasitization levels.

**Figure 1:**
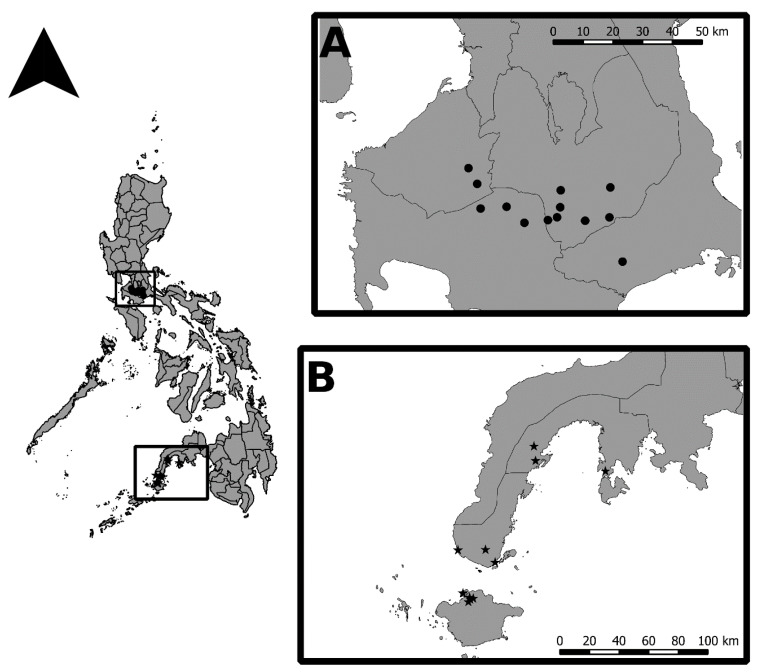
Selected sites in the Philippines with confirmed occurrence of *Aspidiotus rigidus* and *Comperiella calauanica*: (**A**) Southern Tagalog and (**B**) Zamboanga Peninsula.

Field surveys in Southern Tagalog, Luzon Island were conducted on five different dates, August 2014, December 2014, January 2015, June 2015, and June 2016 covering *Aspidiotus rigidus*-affected towns in the Southern Tagalog provinces of Cavite (Silang and Tagaytay), Batangas (Santo Tomas, Talisay, and Tanauan), Laguna (Cavinti, Liliw, Luisiana, Nagcarlan, Pagsanjan, San Pablo, and Sta. Cruz), and Quezon (Candelaria, Lucban, Sariaya, Tayabas, and Tiaong) that were accessible to the trail along the following thoroughfares: Sta. Rosa–Tagaytay Road, Ligaya Drive, Tanauan–Talisay National Road, the Daang Maharlika Highway, and Nagcarlan–Calumpang Road (Figure 1A). In each site (with the exception of Alaminos, Calauan, Los Baños and Rizal in Laguna, and Candelaria in Quezon province where records of occurrences were based on visual observations and reports, and not on actual sampling), a scale-infested coconut tree that had no indication of having been treated with dinotefuran (i.e., coded markings by the PCA, and trunk injection lesions) was selected for periodic sample collection. Leaflet samples from lower fronds were randomly collected from the selected coconut trees that were confirmed to be scale-infested. Samples were collected from the same tree during each sampling period, except when the infested tree had been cut without our notice prior to our field visit. Presence of adult *Comperiella calauanica* in the samples was also recorded.

In the Zamboanga Peninsula, field surveys were conducted in September 2018, January 2019, and January 2020. The survey areas included Zamboanga City, Zamboanga Sibugay, and Isabela City in Basilan Island (which is geopolitically considered part of Zamboanga Peninsula) (Figure 1B). Following the RGA method, two to five trees, regardless of scale infestation status, were randomly selected per sampling point in a survey area.

Mass-reared *C. calauanica* were released initially by the research team in Zamboanga City in November 2016 and January 2017 (average of 22 adult parasitoids in 4 sites situated more than 200 m apart), and subsequently by the PCA in different periods from 2017 to 2018 (100 parasitoids per hectare, per unpublished report) for biological control of *A. rigidus* in all of the survey sites in the Zamboanga Peninsula. These were inoculative releases, as no occurrence of the parasitoid was observed when an infestation of *A. rigidus* was confirmed by preliminary surveys in November 2016 and 2017 in Zamboanga City. In Southern Tagalog, no release of mass reared *C. calauanica* was done because of the observed natural occurrence of the parasitoid during preliminary surveys in April 2014 [18].

### 2.2. Estimation of Field Parasitization Levels

**Figure 2:**
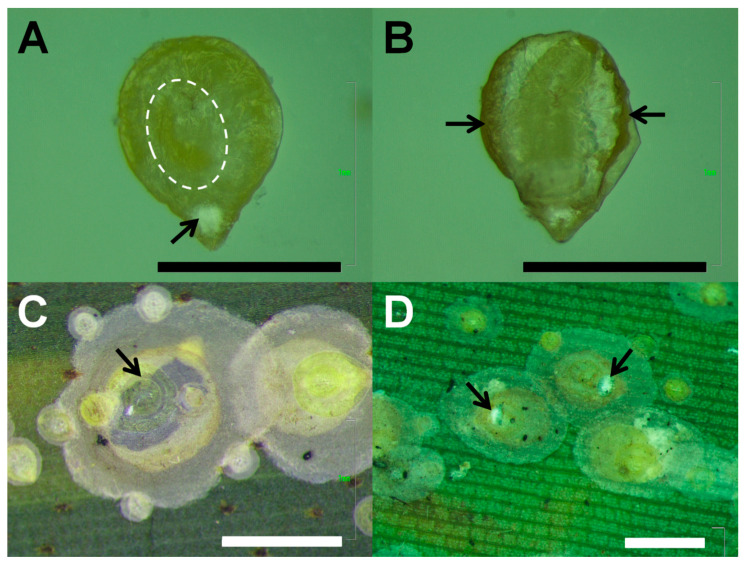
Indicators of parasitization by *Comperiella calauanica* on *Aspidiotus rigidus*: (**A**) white, powdery residue (pointed to by arrow) occurring on ventral pygidium of an isolated scale parasitized by a *C. calauanica* third instar larva (location in the scale indicated by broken oval); (**B**) meconial deposits (pointed to by arrows) produced by the *C. calauanica* fourth instar larva visible in the middle of the isolated, mummified scale; (**C**) dark-colored, *C. calauanica* late pupa inside an intact, mummified scale (pointed to by arrow); and (**D**) exit holes (pointed to by arrows) produced by adult *C. calauanica* upon emergence from their mummified hosts. Scale bars represent 1 mm.

Four samples of *A. rigidus*-infested leaflets collected from the survey areas in Southern Tagalog were assessed per sampling point per sampling period. For standardized and more efficient counting, each sample was subdivided into three subsamples, each being a 10 cm-long leaflet segment. The lower surface of each leaflet segment was then examined under a dissecting microscope for insect counting. The number of live, encrusted *A. rigidus* third instar and mature females with or without indications of parasitization by immature stages (larva and pupa) of *C. calauanica* as well as that of dead scales with exit holes were counted and recorded. Among the parasitization marks noted on live nymphs or mature scales were the presence of a distinctive accumulation of a white powdery residue at the ventral surface of the scale’s pygidium (Figure 2A), the marginal darkening due to the meconial deposition by the parasitoid larva (Figure 2B), and visually detectable manifestation of a late-stage larva or pupa (Figure 2B,C). Destructive counting was done not only to avoid double counting of scales but also to confirm the presence of early-stage larvae which are not readily detectable by simple external inspection of the host even with its scale cover removed.

For the samples from the Zamboanga Peninsula areas which were collected and processed following the RGA method, a 15.24 cm segment was obtained from one random leaflet each from the basal, middle, and apical portions of each of two opposite fronds from a sampled tree, for a total of twelve subsamples per tree. Five trees per site were sampled when possible, for three sampling periods representing the years 2018, 2019, and 2020. A two-dimensional image of the lower surface of each leaflet segment was captured using a high resolution flatbed scanner (Canon 9000F Mark II) (Canon Inc., Tokyo, Japan) for ex-situ insect counting with the aid of ImageJ. In each sample image, the number of presumably live, encrusted *A. rigidus* third instar and mature females with or without marks of parasitization by *C. calauanica*, and the number of dead scales with exit holes were counted.

Percent parasitization was computed using the following formula:

$$\%Parasitization=\frac{n_{P}}{n_{P}+n_{NP}}\;\times 100\%$$

where *n_P_* = number of parasitized scales and *n_NP_* = number of non-parasitized scales. For Southern Tagalog, each sample was represented by the average percent parasitization obtained from the three subsamples. For the Zamboanga Peninsula areas of Zamboanga Sibugay, Zamboanga City, and Isabela City, the average percent parasitization for a sample was obtained from twelve subsamples. Table 1 shows the number of leaflet segment samples collected in the *A. rigidus* infestation sites across pertinent sampling periods.

### 2.3. Data Analysis

For each sampling period, the mean percent parasitization values were compared across points by single-factor analysis of variance (ANOVA), with post-hoc Tukey HSD test when necessary. Correlation with linear regression was performed on the log-transformed number of parasitized scales against the log-transformed total number of scales to determine possible host density dependence of parasitization by *C. calauanica* on *A. rigidus* in Southern Tagalog. 

## 3. Results

### 3.1. Distribution of A. rigidus and C. calauanica in Southern Tagalog Region Survey Sites

**Figure 3:**
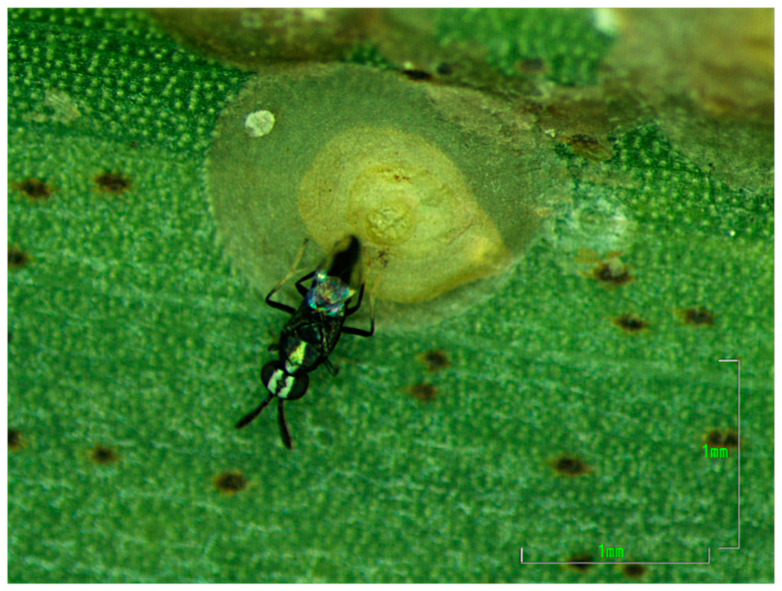
Adult female *Comperiella calauanica* ovipositing on a third instar female *Aspidiotus rigidus* nymph.

Occurrence data in Southern Tagalog, Luzon Island of *C. calauanica* and *A. rigidus* (Table 2) further support their direct relationship, as live *C. calauanica* adults were always found in samples with colonies of *A. rigidus* in all survey sites. Oviposition by adult female parasitoids was also observed during sample processing in the laboratory (Figure 3).

### 3.2. Field Parasitism of C. calauanica on A. rigidus in Selected Sites in Southern Tagalog Region, Luzon Island and in the Zamboanga Peninsula, Mindanao Island

**Figure 4:**
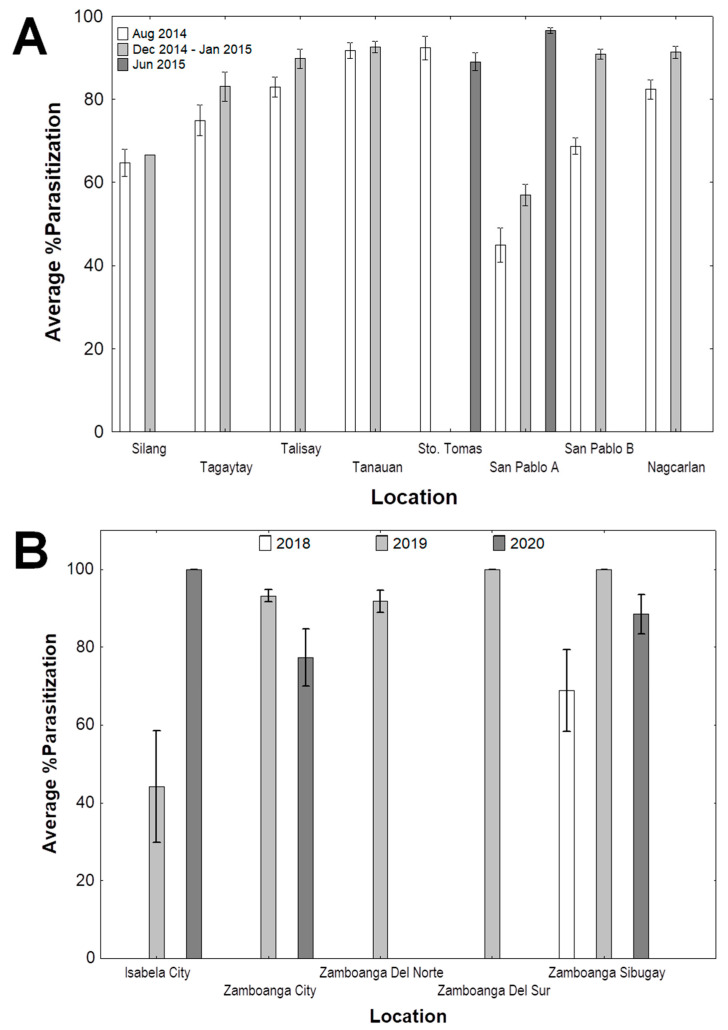
Levels of parasitization (mean ± SEM) of *Aspidiotus rigidus* by *Comperiella calauanica* in the field across survey points in Southern Tagalog (**A**) and in provinces in the Zamboanga Peninsula (**B**) for three sampling periods.

Figure 4A shows the percent parasitization of *A. rigidus* by *C. calauanica* across three different sampling periods: namely, August 2014, December 2014 to January 2015, and June 2015 (Figure 4A). Survey results in August 2014 showed average percent parasitization with means that ranged from 44.93 ± 4.06% in Site A in San Pablo, Laguna (San Pablo A) to 92.38 ± 2.82% in Santo Tomas, Batangas. With the exception of San Pablo A, all the survey points had percent parasitization values higher than 50%. During the period from December 2014 to January 2015, mean average percent parasitization across points ranged from 56.99 ± 2.52% in San Pablo A to 92.60 ± 1.44% in Tanauan, Batangas. A marked increasing trend in the average percent parasitization values in San Pablo A across three sampling periods was recorded, with significant increases between two successive sampling periods until the mean value reached 96.54% in June 2015. Colonies of *A. rigidus* could no longer be found in any of the areas that were sampled by the time of the final surveillance in June 2016. For the same period (December 2014 to January 2015) in Silang, only one set of coconut leaf samples exhibited *A. rigidus* infestation. The rest of the leaflet samples had *A. destructor* and thus, were not included in the analysis. *A. rigidus* seemed to have been displaced by a population of *A. destructor* together with its predator *Telsimia nitida* Chapin (Coleoptera: Coccinellidae). In June 2015, only two among the eight sampling points still had *A. rigidus* colonies on which *C. calauanica* parasitism could be assessed, namely Santo Tomas, with mean percent parasitization of 89.01 ± 2.17%, and San Pablo A with 96.54 ± 0.62%.

For Zamboanga Peninsula provinces, while counting of parasitized and non-parasitized insects was done differently using high resolution scans of leaflet segments instead of actual specimens, percent parasitization was computed similarly to Southern Tagalog. Average percent parasitization values recorded in Isabela City, Zamboanga City, Zamboanga Del Norte, Zamboanga Del Sur, and Zamboanga Sibugay from 2018, 2019, and 2020 are shown in Figure 4B. In 2018, we were able to collect samples from Zamboanga Sibugay only, with an over-all percent parasitization of 68.75 ± 10.49% in all survey points. In 2019, percent parasitization was found to be significantly lower (*p* = 0.0000) in Isabela City (44.22 ± 14.37%) compared to the other provinces (93.21 ± 1.63% in Zamboanga City, 91.79 ± 2.82% in Zamboanga Del Norte, and 100.00 ± 0.00% both in Zamboanga Del Sur and Zamboanga Sibugay). In 2020, percent parasitization was found to be comparable (*p* = 0.1250) for Isabela City (100.00 ± 0.00%), Zamboanga City (77.28 ± 7.42%), and Zamboanga Sibugay (88.47 ± 5.14%). No percent parasitization was obtained for Zamboanga Del Sur, as collected samples either no longer had *A. rigidus* or were infested by *A. destructor* and/or other hemipteran pests. A significant increase in percent parasitization was noted in Isabela City (*p* = 0.0269), and while percent parasitization appears to have decreased between 2019 and 2020 in Zamboanga City and Zamboanga Sibugay, no significant difference was found in both provinces (*p* > 0.05).

### 3.3. Putative Host Density Dependence of Parasitism by C. calauanica Number

**Figure 5:**
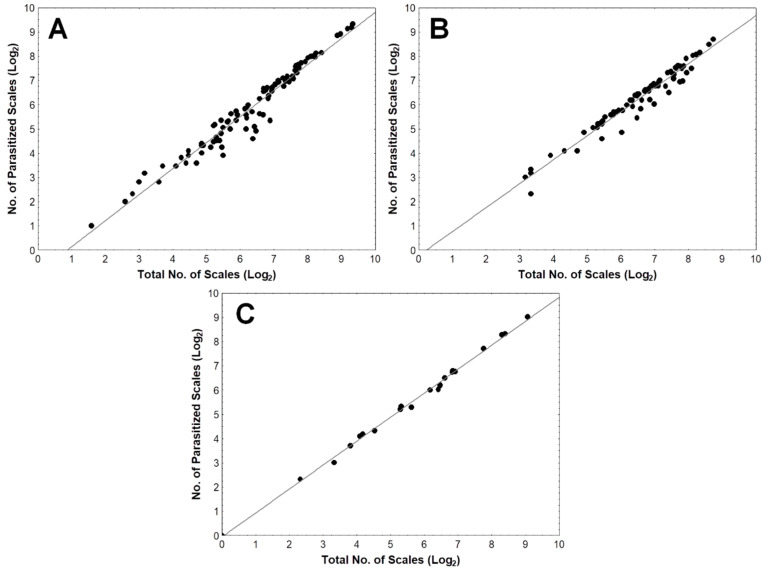
Correlation and regression of the number of parasitized scales against the total number of scales across the three sampling periods: (**A**) August 2014 (r = 0.9764, r^2^ = 0.9534, *p* = 0.0000); (**B**) December 2014 to January 2015 (r = 0.9764, r^2^ = 0.9535, *p* = 0.0000); (**C**) June 2015 (r = 0.9988, r^2^ = 0.9976, *p* = 0.0000).

Putative dependence of *C. calauanica* parasitism on host density in the field was estimated by linear correlation and regression analyses of the number of parasitized *A. rigidus* (n_P_) against the total of scales (n_T_) as respective measures of parasitism and host density. Highly significant, nearly perfect positive correlations (*p* < 0.01) were found between n_P_ and n_T_ for August 2014 (r = 0.9764), December 2014 to January 2015 (r = 0.9764), and June 2015 (r = 0.9988) across the sampling points in Southern Tagalog (Figure 5). The best-fit line does not intersect the x-axis at 0, most observable particularly in the plots for August 2014 and December 2014 to January 2015. The putative host density dependence of parasitism by *C. calauanica* in the field was similarly observed in the Zamboanga Peninsula, particularly in Zamboanga City and in Zamboanga Sibugay, where a number of coconut trees were noted to retain very small patches of *A. rigidus* colonies on a few leaflets and with relatively low abundance of *C. calauanica* adults.

### 3.4. Impact of Parasitization by C. calauanica on A. rigidus Populations and Recovery of Coconut Trees

**Figure 6:**
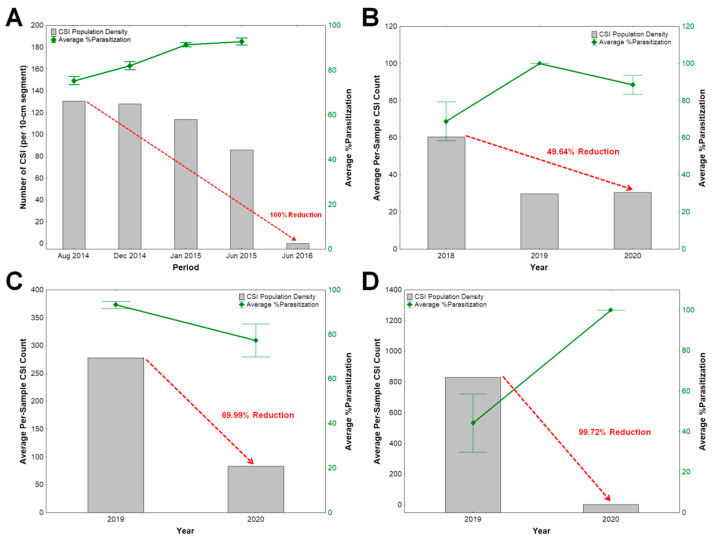
Population density of *Aspidiotus rigidus* and percent parasitization by *Comperiella calauanica* over time in: (**A**) Southern Tagalog from August 2014 to June 2016; (**B**) Zamboanaga Sibugay from 2018 to 2020; (**C**) Zamboanga City, and (**D**) Isabela City from 2019 to 2020. Columns represent averages of pooled scale insect population counts from all the trees sampled for a particular period.

The abundance of *A. rigidus* in this study’s sampling points in Southern Tagalog was observed to have decreased down to zero, suggesting 100% reduction in population density, in a span of only a little more than two years, from the peak of infestation in 2014 up to 2016 (Figure 6A). Gradual recovery from *A. rigidus* infestation of the periodically sampled trees as well as other trees in the surrounding stands of coconut trees in Southern Tagalog was observed during the same duration.

In Zamboanga Sibugay, Mindanao Island, as high as 100% parasitization by *C. calauanica* was noted to have possibly contributed about 50% reduction of *A. rigidus* abundance from 2018 to 2020 (Figure 6B). Infestation by the diaspidid in this province was observed not to have reached outbreak levels as observed in Southern Tagalog and in the neighboring Zamboanga City. Higher reduction in population density of *A. rigidus* was observed in Zamboanga City at about 70% (Figure 6C) and in Isabela City with close to 100% (Figure 6D), in which scale colonies in both areas were determined to be highly parasitized by *C. calauanica*. Recovery from *A. rigidus* infestation of the monitored coconut trees was also observed between 2018 and 2020, parallel to the recorded reduction in scale population density.

## 4. Discussion

Results of this study showed the direct association between *C. calauanica* and *A. rigidus*. This is further confirmed by a series of host-range tests in the laboratory (choice and no choice tests), introducing *C. calauanica* to other related diaspidid species to determine their suitability as alternative hosts (unpublished data). No alternative host has been found so far in all of our subsequent field monitoring and surveys. Assessment of field parasitism of *C. calauanica* on *A. rigidus* in Southern Tagalog showed very high average percent parasitization values that were above 50 percent in all but one of the sampling sites and periods, which was San Pablo A in August 2014. In December 2014 to January 2015, San Pablo A was likewise found to have the lowest mean average percent parasitism of 56.99%, significantly lower compared to all other points. It was observed that the tree from which samples were periodically collected in this particular site was constantly subjected to smoke and dust from the combustion of coconut shells used as fuel for outdoor cooking. It is likely that the smoke and dust provided a disturbance that could have affected the rate of establishment of *C. calauanica* on the colonies of *A. rigidus* in the sampled tree. Dust has been described as an environmental disturbance that may cause disruptions in the activity of parasitic Hymenoptera, or even lead to population elimination [20]. High percent parasitization values were also recorded in the Zamboanga Peninsula sites from 2018 to 2019, the average of which were found to have reached up to 100% in Zamboanga Sibugay in 2019 and 100% in Isabela City in 2020. These results suggest that *C. calauanica* was able to establish in the areas surveyed at a considerably high degree.

It must be noted that the percent parasitization values obtained in this study could have underestimated the actual parasitism of *C. calauanica* across the sampling points, given the fact that we were unable to count as parasitized the scales that possibly already contained *C. calauanica* eggs. Nevertheless, the very high degree of parasitization levels, observed and quantitatively estimated, throughout the sampling points, and across sampling periods suggest the efficacy and efficiency of *C. calauanica* as natural enemy of *A. rigidus*. This endoparasitoid has been reported to be thelytokous [18,19], and its efficiency against *A. rigidus* could possibly be attributed to its mode of reproduction.

Results of correlation and regression analyses of data from Southern Tagalog suggest that the number of parasitized *A. rigidus* has a very high tendency to increase with the total number of scales in a nearly perfectly linear manner. By implication, parasitism by *C. calauanica* would also have a very high tendency to increase with host density. The scatterplots in Figure 5 shows regression lines that intersect with the x-axis (representing the total number of hosts) at non-zero values, suggesting a positively host density-dependent parasitism that requires the host, *A. rigidus*, to be present at a particular density before *C. calauanica* would parasitize. This would consequently imply that, barring other mortality factors, populations of *A. rigidus* at low densities may still remain in recovered coconut trees, with the parasitoid occurring in low abundance. A mathematical model by Palen et al. [21] assumed that the parasitoid may exhibit Holling type III functional response, in which parasitization on an alternate host is necessary for survival in the absence of the primary host. However, our studies in the field, including ones subsequent to the current, have yet to find such an alternate host for *C. calauanica*. Without an alternate host, it is likely that *C. calauanica* populations could be maintained with small patches of *A. rigidus* colonies in the field, as we have observed in a number of coconut palms in Pagbilao, Quezon in 2017, and in seedlings of mangosteen (a differential dicot host of *A. rigidus*) in Tiaong, Quezon in 2016 and 2017.

Host- or prey-density dependence of parasitism or predation could be observed less frequently in field studies than in laboratory studies due to the influence of environmental and biotic disturbances such as precipitation and natural enemies. This characteristic could possibly be common in native, monophagous, solitary species of parasitoids especially on sedentary hosts [22]. However, *C. calauanica* is not only a native, solitary, and highly host-specific parasitoid, but also has no effective natural enemy that has been observed or reported in the field. *Marietta carnesi* (Howard) (Hymenoptera: Aphelinidae), was found in the current study in August 2014, with a single individual appearing among the pool of 96 leaflet segment samples from Southern Tagalog that were examined for insect counting. While the association of *M. carnesi* with *C. calauanica* was not actually established in this study, the former has been reported to be hyperparasitic to the latter [23]. *M. carnesi* has been reported as a secondary parasitoid in coccoids, and hyperparasitic on at least two species of *Comperiella* [17], although it seems that the almost negligible occurrence of this hyperparasitoid did not affect the very high levels of parasitization by *C. calauanica* and the high mortality of *A. rigidus* in Southern Tagalog. The levels of hyperparasitism of *M. carnesi* found in *C. calauanica*-parasitized colonies of *A. rigidus* in Orani, Bataan, Luzon Island in 2015 were found to be very low, and has been suggested not to have much effect on the biological control potential of *C. calauanica* [24]. Additionally, with the exception of typhoon Rammasun in July 2014, there was no episode of significant rainfall or other climatic disturbances in the Southern Tagalog region recorded from 2014 to 2015 when our field surveys and sample collections were done. It is likely due to these reasons that our findings appear to point to a putative host density-dependent parasitization by *C. calauanica* in the field. Parasitic Hymenoptera exhibiting density-dependent parasitism are effective as biological control agents against their hosts [20]. Once the population density of *A. rigidus* has decreased to below the economic injury level, *C. calauanica* can maintain such host density, barring other mortality factors. This is consistent with our observations of very small patches of *A. rigidus* colonies, with few *C. calauanica* adults and immatures, remaining on some leaflets in some of the coconut trees in Zamboanga City and Zamboanga Sibugay that had already completely recovered from what used to be severe infestation by the scale between 2018 and 2020.

Colonies of *A. rigidus* could no longer be found in the sampling points in Southern Tagalog by the time a final surveillance was conducted in June 2016, hence the absence of percent parasitization values for that period. In a span of only two years, from the peak of the outbreak in early 2014 to 2016, the abundance of *A. rigidus* was found in this study to have decreased dramatically down to zero, even without chemical control by trunk injection of dinotefuran. Visible recovery of the periodically sampled coconut trees, as evidenced by flowering, and the increase in the proportion of green, healthy fronds to sickly fronds with chlorotic leaflets, was observed parallel to the recorded decrease in the abundance of *A. rigidus* and the very high percent parasitization of scales by *C. calauanica*. These observations, together with findings of possible host density-dependent parasitism in the field, not only points to the potential of this *C. calauanica* as an effective biological control agent against *A. rigidus*, but also suggests the putative role of this encyrtid in the natural control of the scale insect outbreak in Southern Tagalog.

*C. calauanica* was not released, but was found naturally occurring in the Southern Tagalog region. Our findings of high degrees of parasitism of *C. calauanica* a few months after its discovery in 2014 [18], and throughout 2015 to 2016 towards the decline in the abundance of *A. rigidus* in the field, strongly point to the contribution of natural biological control in the eventual management of the pest outbreak. Although percent parasitism has been regarded as being inadequate to measure the effect of parasitoids on populations of their hosts [25,26], and that the impact of biological control agents is very difficult to assess on the population level [27], we believe that the recovery of the affected coconut trees from the infestation, concurrent to both the high parasitization levels in *A. rigidus* colonies and reduction in the population density of the pest suggests otherwise in the case of *C. calauanica*. *A. rigidus* populations have become very scarce in Southern Tagalog by 2016, and the reduction may have been caused by the consistently high parasitization by *C. calauanica* between 2014 and 2016. It has been suggested that parasitism rates of greater than 50%, when sustained over a wide spatial and temporal range, may reduce populations of affected hosts [28].

An outbreak of *A. rigidus* had been reported in Basilan Island during almost the same time as the outbreak in Southern Tagalog. The closely situated Zamboanga Peninsula was at a considerable risk of *A. rigidus* invasion and outbreak, especially given that the pest is generally believed to be dispersed by wind [3]. We were able to confirm the occurrence of the diaspidid in Zamboanga City in 2017, shortly after which an outbreak began. Inoculative releases of mass-reared *C. calauanica* from Southern Tagalog were carried out by our team and the Philippine Coconut Authority in 2017 as part of the IPM strategy in coconut plantations in the province.

In the province of Zamboanga Sibugay, an infestation buffer zone was identified due to its close vicinity to Zamboanga City. While *A. rigidus* was able to invade some areas in this province, the infestations were not able to reach the same outbreak levels observed in Zamboanga City. This is perhaps due to inoculative releases of *C. calauanica* in 2018 by the Philippine Coconut Authority, which could have maintained the population of *A. rigidus* at manageable levels as suggested by our findings. Recovery of *A. rigidus*-infested coconut trees in our study sites in the Zamboanga Peninsula was observed within a year from inoculative releases of mass-reared *C. calauanica*, without any chemical control measures being implemented, and without the occurrence of any severe weather disturbance in the region. Collectively, our current findings and observations strongly suggest *C. calauanica* parasitism as a major mortality factor that has contributed significantly to the control of *A. rigidus* in the field in the Philippines.

After the cases in Southern Tagalog and Zamboanga Peninsula, outbreaks of *A. rigidus* were reported in coconut plantations in Tablas Island, Romblon (located south of Southern Tagalog) in early 2018 [29,30], and in Albay, Bicol Region (southeast of Southern Tagalog) [29,31] in the same year. In both cases, *C. calauanica* were released for biological control, and the results were reported to be similar to those observed in Southern Tagalog and Zamboanga Peninsula [31]. Consequently, *C. calauanica* has been recognized and accepted in the country as the biological control agent for *A. rigidus*, and has become an important part of the Philippine Coconut Authority’s IPM program for *A. rigidus* [29]. At present, the main rearing facility for *C. calauanica* is in De La Salle University Laguna Campus, with at least two satellite rearing facilities being maintained by the Philippine Coconut Authority in Albay and in Zamboanga City, respectively. Additional satellite rearing facilities may need to be established for quick response to new areas of invasion by *A. rigidus* in the Philippines.

## 5. Conclusions

There has been much debate on the mortality factors that contributed to the management of the *A. rigidus* outbreak in the Southern Tagalog region of Luzon Island, Philippines. *C. calauanica,* which was discovered in the same region, has been found as a very effective, host-specific parasitoid of the diaspidid pest. Its high levels of field parasitism from 2014 to 2015 were found to parallel the decline in the population density of *A. rigidus* and recovery of affected coconut trees that were not treated with dinotefuran. Additionally, during the same period, our results from the region suggested putative host density-dependent parasitism by *C. calauanica* in the field. Release of mass-reared *C. calauanica* as part of the IPM strategy against *A. rigidus* in infested areas in the Zamboanga Peninsula yielded results from 2018 to 2020 similar to those from Southern Tagalog, even without the occurrence of a major climatic disturbance and use of dinotefuran. While our findings in Southern Tagalog may have only suggested the potential of the encyrtid as biological control agent against *A. rigidus*, our results in the Zamboanga Peninsula have validated our claims that parasitization by *C. calauanica* has been a very important mortality factor that contributed to the management of populations of the invasive *A. rigidus* in the Philippines, and that biological control using *C. calauanica* should be a major component of IPM for *A. rigidus* not only in the country but possibly also in other tropical countries where this pest may invade.

## Figures and Tables

**Table 1:** Distribution of leaflet segment samples collected in study sites with confirmed *Aspidiotus rigidus* infestations. Numbers in parentheses indicate the number of segments with *A. rigidus* colonies.

Site	August 2014	December 2014	January 2015	June 2015	June 2016	September 2018	January 2019	January 2020
Cavite								
Silang	12 (12)	12 (3)		12 (0)	12 (0)			
Tagaytay	12 (11)	12 (12)		12 (0)	12 (0)			
Batangas								
Talisay	12 (12)	12 (12)		12 (0)	12 (0)			
Tanauan	12 (12)	12 (12)		12 (0)	12 (0)			
Santo Tomas	12 (12)			12 (0)	12 (0)			
Laguna								
San Pablo A	12 (12)	12 (12)		12 (0)	12 (0)			
San Pablo B	12 (12)	12 (12)			12 (0)			
Nagcarlan	12 (12)		12 (12)	12 (0)	12 (0)			
Zamboanga Peninsula								
Isabela City							12 (11)	120 (6)
Zamboanga City							120 (102)	120 (18)
Zamboanga Del Norte							120 (52)	
Zamboanga Del Sur							120 (33)	72 (0)
Zamboanga Sibugay						120 (41)	60 (34)	84 (6)

**Table 2:** Occurrence of *Aspidiotus rigidus* and *Comperiella calauanica* in select points in Southern Tagalog as observed across the three survey and sampling periods. Presence is indicated by +, while absence is indicated by −.

Point	*Aspidiotus rigidus*	*Comperiella calauanica*
Cavite		
Silang	+	+
Tagaytay	+	+
Batangas		
Malvar	+	+
Talisay	+	+
Tanauan	+	+
Santo Tomas	+	+
Quezon		
Tiaong	−	−
Candelaria	+ *	+ *
Sariaya	−	−
Tayabas	−	−
Lucban	−	−
Laguna		
Calauan	+	+
Alaminos	+	+
Los Baños	+	+
Rizal	+	+
San Pablo	−	−
Luisiana	−	−
Cavinti	−	−
Pagsanjan	−	−
Sta. Cruz	−	−
Liliw	−	−
Nagcarlan	+	+

* Occurrence reported in mangosteen prior to August 28 survey.
